# P-2143. Colonization with Fluoroquinolone-Resistant Streptococcus mitis-oralis Group Prior to Hematopoietic Cell Transplantation and Subsequent Streptococcal Bacteremia

**DOI:** 10.1093/ofid/ofaf695.2306

**Published:** 2026-01-11

**Authors:** Lee Gottesdiener, Supram Hosuru Subramanya, Jamie Marino, Juliet Barker, Tsiporah Shore, Rosemary Soave, Markus Plate, John Lee, Barry N Kreiswirth, Cazer Casey, Lars Westblade, Michael J Satlin

**Affiliations:** Weill Cornell Medicine, New York, New York; Weill Cornell Medicine, New York, New York; Weill Cornell Medicine, New York, New York; Weill Cornell Medicine, New York, New York; Weill Cornell Medical College/ New York Presbyterian Hospital , New York; Weill Cornell Medicine, New York, New York; Center for Discovery and Innovation, Hakensack Meridian Health, Nutley, New Jersey; College of Veterinary Medicine, Cornell University, Ithaca, New York; Weill Cornell Medicine, New York, New York; Weill Cornell Medicine, New York, New York

## Abstract

**Background:**

Bacteremia due to viridans group streptococci (VGS), particularly *Streptococcus mitis-oralis* group (SMOG), is common in neutropenic patients after hematopoietic cell transplantation (HCT) despite use of fluoroquinolone (FQ) prophylaxis. VGS bloodstream isolates in patients receiving FQ prophylaxis are typically FQ-resistant (FQ-R). This study assessed the prevalence of colonization with FQ-R SMOG prior to HCT and the risk of VGS bacteremia during neutropenia.

**Table 1.**
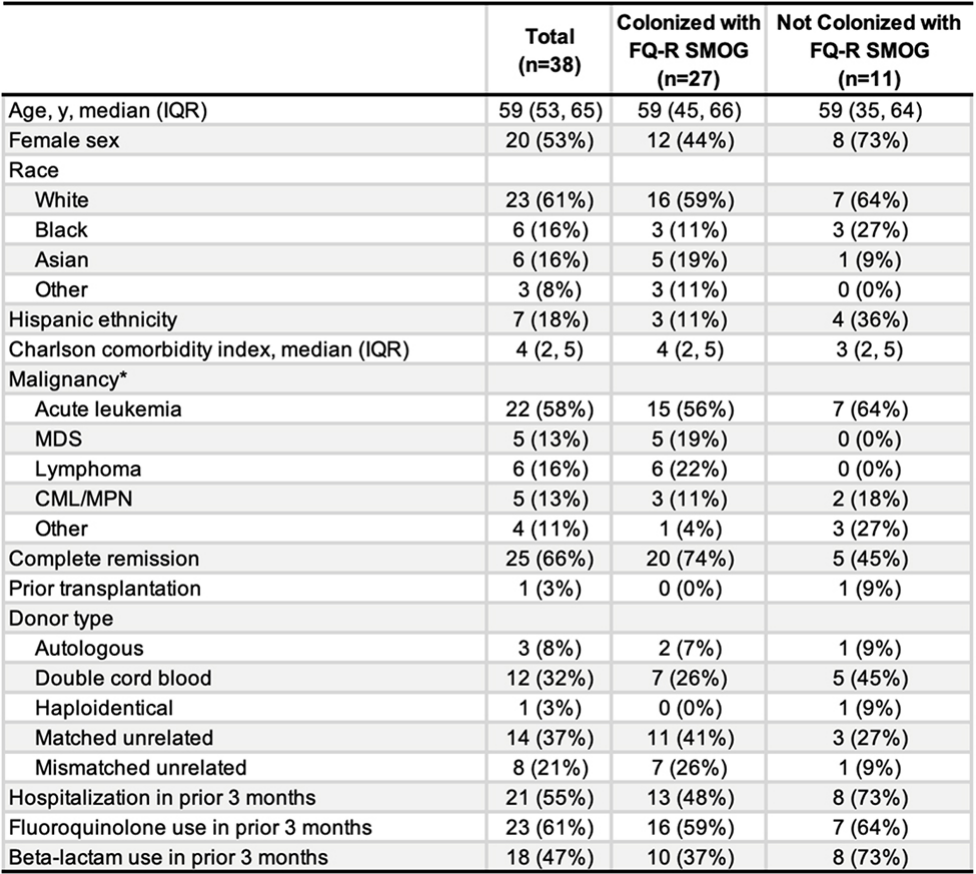
Baseline characteristics of patients, with stratification by colonization with FQ-R SMOG at enrollment *Patients may have more than one malignancy, therefore totals may exceed 100%. Abbreviations: CML, Chronic myeloid leukemia; IQR, Interquartile Range; MDS, Myelodysplastic syndrome; MPN, Myeloproliferative neoplasm; FQ-R SMOG, Fluoroquinolone-resistant Streptococcus mitis-oralis group.

**Figure 1.**
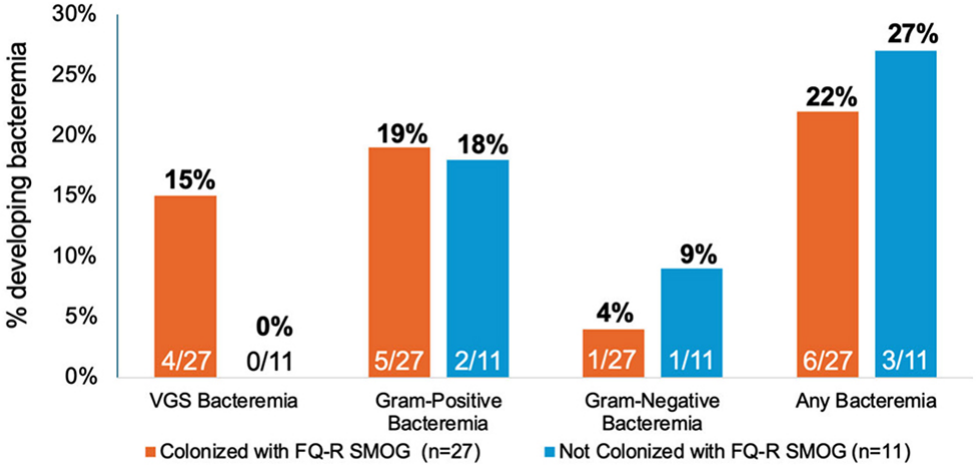
Proportion of patients developing VGS bacteremia, Gram-positive bacteremia, Gram-negative bacteremia, and any bacteremia during neutropenia by FQ-R SMOG colonization status. P values represent comparisons between patients with and without FQ-R SMOG colonization using Fisher’s exact test. Organisms causing non-VGS bacteremia included coagulase-negative Staphylococcus, Enterococcus faecium, Escherichia coli, and Gemella haemolysans. Abbreviations: FQ-R, Fluoroquinolone-resistant; SMOG, Streptococcus mitis-oralis group; VGS, Viridans group streptococci

**Methods:**

This is an interim report of a prospective observational study enrolling patients undergoing HCT at a New York City hospital from June 2024 to February 2025. We collected buccal, oropharyngeal, and perianal swabs (ESwabs) during the week prior to HCT. Swabs underwent selective culture by direct plating on Columbia nalidixic acid agar with levofloxacin (LVX) disks and by broth enrichment (tryptic soy broth with 2 μg/mL of LVX and 4 μg/mL of colistin) before plating. LVX resistance was confirmed by disk diffusion and species identification was determined by matrix-assisted laser desorption ionization–time of flight mass spectrometry. Patients received LVX prophylaxis during neutropenia and were followed until neutrophil recovery or up to 30 days.

**Results:**

Among the first 38 patients enrolled, 35 (92%) underwent allogeneic HCT and 23 (61%) had received FQs within 3 months of enrollment (Table 1). Prior to HCT, 27 (71%) were colonized with FQ-R SMOG, and an additional 7 (18%) of remaining patients were colonized with FQ-R VGS species other than SMOG. Four (15%) patients with FQ-R SMOG colonization developed VGS bacteremia (all due to SMOG) compared to none without FQ-R SMOG colonization (Figure 1; p=0.3). Median time from HCT to VGS bacteremia was 6 days (interquartile range [IQR] 6-9). Compared to FQ-R SMOG-colonized patients without VGS bacteremia, those with VGS bacteremia were more likely to have previously lost ≥5 teeth (p=0.013) and had a longer median duration of neutropenia (21 vs 14 days; p=0.033).

**Conclusion:**

Colonization with FQ-R SMOG prior to HCT is common. In the setting of FQ prophylaxis, VGS bacteremia occurs primarily in colonized patients and is likely caused by their colonizing FQ-R strain. Screening for FQ-R SMOG prior to HCT may identify patients at risk of VGS bacteremia despite FQ prophylaxis.

**Disclosures:**

Markus Plate, MD, F2G: Grant/Research Support|Gilead: Grant/Research Support|Merck: Grant/Research Support John Lee, MD, Astellas: Honoraria|BioFire Diagnostics, LLC: Grant/Research Support|Calliditas: Travel support|Eurofins Viracor: Patent US-2020-0048713-A1 licensed to Eurofins Viracor Lars Westblade, PhD, Elements Materials Technology: Grant/Research Support|Hardy Diagnostics: Grant/Research Support|Melinta Therapeutics: Grant/Research Support|Selux Diagnostics: Grant/Research Support|Shionogi: Advisor/Consultant|SNIPRBIOME: Grant/Research Support Michael J. Satlin, MD, MS, AbbVie: DSMB participant|bioMerieux: Grant/Research Support|Merck: Grant/Research Support|SNIPRBiome: Grant/Research Support

